# Wire Syndrome Associated with Multistrand Orthodontic Retainers: A Proposed Etiopathogenic Concept

**DOI:** 10.3390/dj14090540

**Published:** 2026-08-31

**Authors:** Sophie-Myriam Dridi, Carole Charavet

**Affiliations:** 1 Unité de Parodontologie, Pôle IMBD, CHU de Nice, Département de Parodontologie, Faculté de Chirurgie Dentaire, Université Côte d’Azur, 06100 Nice, France; 2 Unité d’Orthopédie Dento-Faciale, Pôle IMBD, CHU de Nice, Département d’Orthopédie Dento-Faciale, Faculté de Chirurgie Dentaire, Université Côte d’Azur, 06100 Nice, France

**Keywords:** wire syndrome, fixed orthodontic retainer, multistranded wire, etiopathogenesis, orthodontic biomechanics, cohesive failure, unwanted tooth movement

## Abstract

**Background/Objectives**: Wire syndrome (WS) is a recognized post-orthodontic complication associated with bonded fixed retainers. It is characterized by abnormal, unexpected or excessive tooth movement in teeth that are still being held in place by a wire retainer. WS is likely to be underdiagnosed due to its slow, often asymptomatic, progression in the early stages, as well as due to a lack of knowledge among practitioners. This article proposes an etiopathogenic concept for WS associated with twisted or braided multistrand wires, with the aim of improving our understanding of the mechanisms involved in its onset and iatrogenic progression for the purposes of prevention and clinical management. **Materials and Methods**: A narrative review of the available clinical and experimental data in the literature was conducted to identify factors that could be involved in the initiation and progression of WS. **Results**: The qualitative analysis suggested that WS is the result of a multifactorial phenomenon that combines intrinsic factors linked to the mechanical properties of the orthodontic retainer and extrinsic factors corresponding to functional, muscular, and parafunctional stresses exerted on the teeth. Several etiopathogenic scenarios can be proposed, including loss of wire–composite adhesion, maintenance of this adhesion in the context of excessive muscular forces, and a mixed mechanism. In all cases, secondary deformation of the wire could alter its mechanical properties progressively, leading to unintended orthodontic activation of the appliance. This review also emphasizes the importance of rigorous retainer placement protocols, prolonged post-orthodontic follow-up and preventing excessive mechanical stress. Artificial intelligence could be a promising way to detect WS early on through the automated analysis of successive optical impressions. **Conclusions**: This review puts forward a coherent etiopathogenic concept for WS associated with multistrand wires. This concept is based on the interaction between wire deformation, progressive alteration to the wire–composite interface and mechanical stresses in the oral environment. This concept could explain the wide clinical variability of the syndrome, as well as its often slow and insidious progression. A better understanding of these mechanisms is essential for improving the prevention, early diagnosis, and clinical management of WS.

## 1. Introduction

Wire syndrome (WS) is now recognized as a post-orthodontic complication associated with fixed retainers. It has been defined by Charavet et al. [1] as follows: “Fixed orthodontic retainers can provoke aberrant, unexpected, unwanted, or unexplained tooth movement on teeth still bonded by a fixed retainer placed after orthodontic treatment, which could induce progressively iatrogenic dental and periodontal complications, functional and/or esthetic, ranging from minor teeth displacement to teeth expulsion from the bone with loss of vitality. In the presence of severe WS, the retainer may become detached or fractured. WS is not a classic orthodontic relapse, and the position of the teeth does not correspond to any previous situation” [1]. Wire syndrome should therefore be clearly distinguished from other complications associated with fixed retainers-for example, partial or total debonding of the retainer wire, its fracture, or retainer failure more broadly-which may themselves lead to a classic orthodontic relapse. The latter corresponds to a return of the teeth toward their pretreatment position, whereas WS produces tooth positions that do not correspond to any prior situation, on a wire that remains well bonded and unfractured (except in extreme end-stage cases).

Its prevalence varies considerably depending on the population studied, ranging from 1.1% to 43% [1,2].

WS progresses gradually and insidiously, potentially leading to significant dento-periodontal consequences, as well as esthetic alterations that can be severe [3].

**Figure 1:**
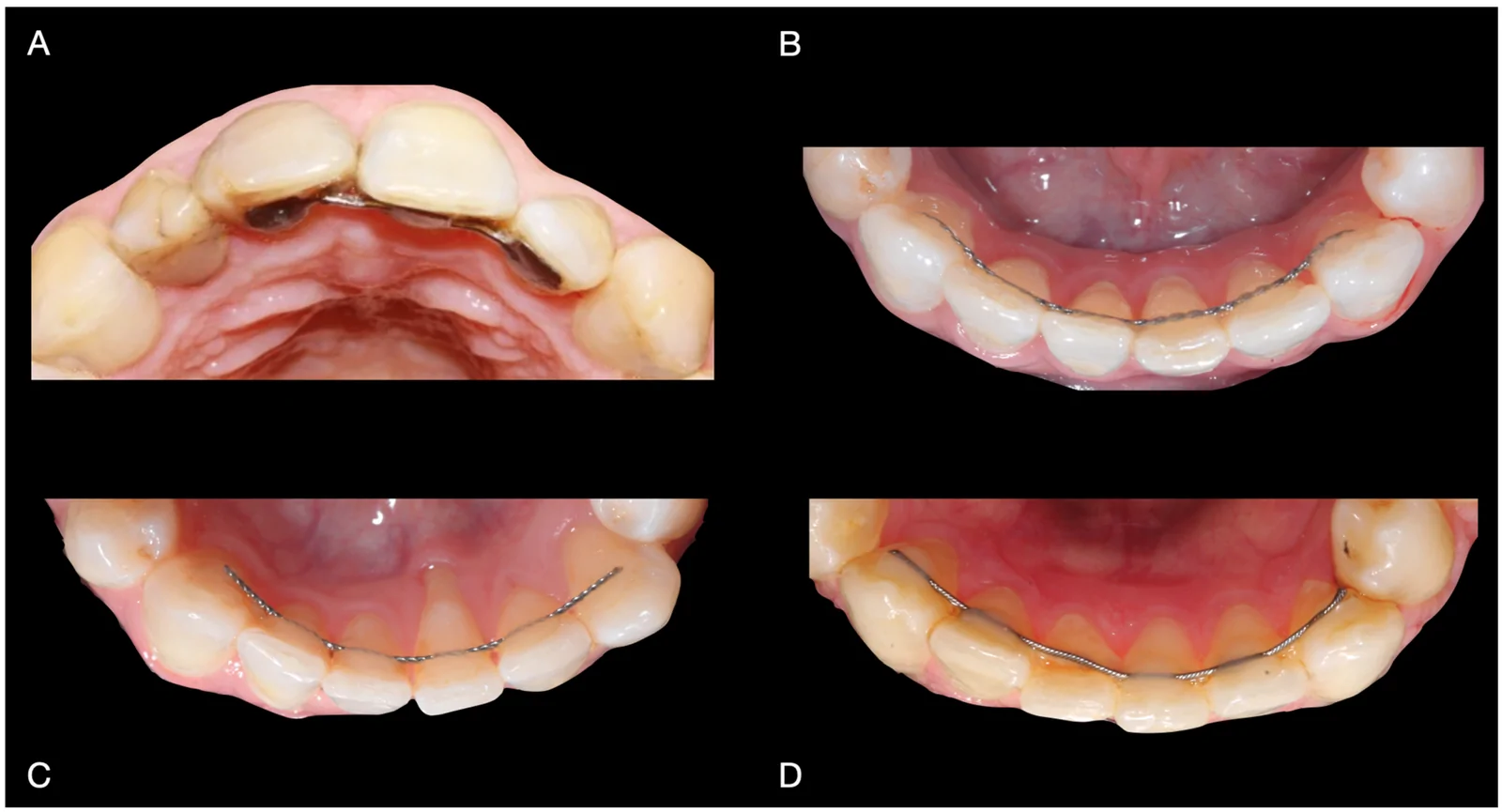
Intraoral views illustrating wire syndrome associated with four different fixed orthodontic retention–wire configurations in the maxillary arch (**A**) and mandibular arch (**B**–**D**). Photos courtesy of the authors: Prof. Sophie-Myriam Dridi and Prof. Carole Charavet.

Clinically, the syndrome has been reported in connection with various types of retention wire, including single strand, multistrand, braided, twisted, flat and round wires, regardless of their diameter or surface treatment (Figure 1). An international Severity-Based Classification of Wire Syndrome was developed and provided a reliable and reproducible tool applicable across dental specialties and easily integrated into routine retention follow-up using three photographs, facilitating systematic early detection of WS [4].

Multistrand wires are widely preferred among these wires. They meet the main requirements for fixed retainers, including ease of use, stabilization of the teeth following active orthodontic treatment, resistance to functional stresses without breakage or deterioration, and the ability to accommodate physiological tooth movements [5,6,7].

Nevertheless, these appliances also have certain limitations. Compared to removable retainers, they are more difficult to maintain and require regular and prolonged clinical follow-up [8]. However, this follow-up is not always adhered to in daily practice [9]. Furthermore, bonded wires may suffer partial detachment, fractures, or be associated with the development of WS.

Although the majority of orthodontists are now aware of this WS, many report not having clearly established strategies to prevent or limit its occurrence [10].

Several risk factors have been suggested in the literature to explain the onset of this syndrome in cases involving multistrand wires [11,12].

These factors include:

- An inadequate fitting protocol (e.g., inaccuracy in shaping the wire and/or adapting it to the tooth surfaces);
- Repeated external mechanical stress, such as traumatic use of interdental hygiene devices, nail-biting, teeth-grinding or ultrasonic vibrations during scaling;
- Excessive masticatory or muscular forces that are insufficiently compensated for.
- However, the exact circumstances surrounding the onset of WS associated with multiwire retainers remain poorly understood.

A better understanding of the mechanisms involved in the onset and progression of this syndrome would enable therapeutic strategies to be optimized. It would also address the recurring questions of practitioners (including general dentists, periodontists and orthodontists) who are responsible for its management. For example, should the fixed retainer be removed as soon as WS is diagnosed? Is it necessary to remove the wire before periodontal treatment? Can a fixed retainer be repaired or re-bonded? How can the risk of WS be minimized when a new fixed retainer is required?

Furthermore, identifying the etiopathogenic mechanisms of WS would improve manufacturing and bonding protocols for fixed retainers while shedding light on potential links between this syndrome and other types of retainer wire. It would also allow us to provide patients with more personalized support based on reliable scientific data, which is becoming increasingly important to them [13]. Finally, it would open new avenues for research in the clinical, biomechanical, and biotechnological fields.

The main objective of this narrative review is therefore to critically analyze the mechanisms potentially involved in WS associated with bonded fixed retainers made using twisted or braided multistrand wires.

## 2. Search Strategy, Evidence Selection and Evidence Synthesis

The search strategy focused on studies examining the failure of post-orthodontic retention appliances. All study types were eligible, including in vitro, cross-sectional, retrospective, prospective and case series studies, except for animal studies, narrative reviews and expert reports. The following terms were used to search the PubMed/MEDLINE, Embase and Cochrane Library databases with no restrictions on language or date: ‘wire syndrome’, ‘orthodontic wire’, ‘bonded retainer’, ‘fixed orthodontic retainers’, ‘wire composite interface’, ‘failure’. A manual search was also carried out using the references cited in the studies selected from the databases.

Ultimately, mainly due to the methodological constraints, very few studies provided insight into the mechanisms of WS, with only 10 in vitro studies, one retrospective study and one cross-sectional study being conducted. Nevertheless, the collected data made it possible to identify two broad categories of factors that are likely to play a role in the development of WS.

### 2.1. Intrinsic Factors Related to the Properties of the Retention Wire

Several mechanisms of fixed retainer failure have been identified in the literature. These include adhesive failure at the enamel–composite interface, cohesive failure at the wire–composite interface, and a combination of the two. Another mechanism is wire fracture under stress [14,15].

In the case of wire syndrome (WS), where the composite is assumed to remain bonded to the tooth surfaces, only cohesive failure-corresponding to a total or partial breakdown of the micromechanical interface between the wire and the composite-appears consistent with the occurrence of this syndrome.

To assess the quality of this bond, several in vitro experimental protocols have been developed to analyze the behavior of retention wire subjected to mechanical stresses (Table 1).

Tensile tests assess the mechanical strength of the wire–composite assembly when subjected to an axial tensile load. Conversely, deformation or bending tests analyze the wire’s ability to withstand permanent deformation without detaching from or breaking the composite.

While these experiments do not accurately replicate the stresses exerted on the retainer in vivo, the results still contribute to a better understanding of these fixed retainers’ failure mechanisms.

The study by Bearn et al. (1997), conducted on three- or six-strand twisted or braided metal wires subjected to tensile cycles, was one of the first to demonstrate the impact of surface condition on adhesion to composite materials [16]. The authors also demonstrated that abrasion of the composite pads surrounding the wires increased the risk of failure at the wire–composite interface, thereby emphasizing the importance of local mechanical interactions [16].

These observations were supported by the work of Baysal et al. (2012), who showed that the mechanical properties of the wire affect the strength of the wire–composite interface [19]. Unlike stiffer wires, flexible wires were more likely to deform between the composite posts under vertical forces, presenting an increased risk of fracture rather than composite detachment [19].

For their part, Paolone et al. (2015) emphasized that the quality of adhesion between the wire and the composite depended on the mechanical properties of the entire retainer [20]. Their results showed that gold-plated twisted round wires or braided rectangular wires embedded in a micro-nano-filled composite performed best, suggesting a synergy between the nature of the wire and the composition of the composite [20].

The study by Milheiro et al. (2015), which was based on a finite element analysis, provided further insight into these observations [6]. The authors demonstrated that the most critical area of the fixed retainer was the wire–composite interface rather than the composite–enamel interface. They also showed that stress distribution in this region depended on the length of free wire between the composite posts. When the posts were reduced in the mesio-distal direction, stresses were concentrated mainly around the wire. The authors concluded that a critical post size of between 4 and 2 mm would be necessary to ensure optimal adhesion between the wire and the composite [6].

Finally, a study by Kurnaz et al. (2025) showed that the bond strength of a metal wire to composite, as well as the wire’s deformability, depended more on the metal’s nature than on its surface treatment [24]. The authors recommend stainless steel wires over nickel-titanium wires in situations involving high occlusal forces or an unfavorable periodontal context, as the former are more flexible and can deform more easily [24].

### 2.2. Extrinsic Factors Related to Trauma and Forces Exerted on the Teeth

Several clinical and in vitro experimental studies highlight the potential role of extrinsic factors in the development of WS.

#### 2.2.1. Clinical Studies

A retrospective case–control study by Verschueren et al. (2023) revealed a strong link between the short-term failure of bonded fixed retainers and specific parafunctional habits [25]. All the patients wore the same type of bonded post-orthodontic retainer, made using a six-strand twisted stainless steel wire, from canine to canine in the maxilla and/or mandible. The results showed that failures occurred more frequently in the mandible, particularly near the midline (sectors 31–41). In the maxilla, however, failure sites were more widely distributed across the entire anterior region. In most cases, failure involved a single tooth (62.14%), while in 37.9% of cases, it involved two or more teeth. Furthermore, a significant association was found between nail-biting and lingual dysfunction, and the failure of mandibular retainers (*p* < 0.001 and *p* = 0.002, respectively). Regarding maxillary fixed retainers, only a significant association between lingual dysfunction and retainer failure was observed (*p* = 0.021) [25].

In a cross-sectional study of young adults, Charavet et al. (2024) demonstrated a correlation between mandibular WS and the presence of a deep labio-mental fold, which is considered one of the clinical signs of excessive muscle strength in the chin region (OR: 16.5; 95% CI: 1.9–146.8; *p* = 0.01) [2].

#### 2.2.2. In Vitro Studies

Several in vitro studies have also demonstrated that teeth held in place by a fixed retainer can be displaced if sufficient force is applied to the retainer itself (Table 1).

The study by Cooke and Sherriff (2010) is also relevant to this framework [17]. The authors assessed the bond strength required to detach a multistrand retention wire from acrylic resin models. They found that the average bond strength required to break the cohesive bond at the wire–composite interface ranged from 37.7 N to 41.4 N, depending on the type of wire. They thus suggested that when a vertical force is applied to the retainer, stresses are applied to the free segment of the wire, leading to its deformation and ultimately a cohesive failure of the wire–composite interface. In a second experimental phase, the authors also demonstrated that reusing a wire that had previously been detached to create a new retainer reduced the bond strength [17]. This suggests a deterioration in the wire’s mechanical performance in this context.

These observations are complemented by the study of Sifakakis et al. (2011) [18]. The authors applied vertical and horizontal forces to acrylic resin models comprising six teeth (from canine to canine), which were connected by a multistrand wire retainer. This was done to induce displacement of the free segment of the wire. They found that wire displacement of just 0.2 mm generated significant forces on the teeth: 1 N in the vertical direction and 1.5 N in the horizontal direction [18]. Such forces could potentially induce undesirable tooth movements, particularly intrusion, extrusion and tooth inclination.

Similar findings were reported in the study by Samson et al. (2018) [21]. The authors assessed the bond strength and deformation under load of three types of multistrand retention wire. Occlusal and flexural forces were applied to the free ends of the wires incorporated into the experimental models. The results showed that the average adhesion force required to detach the twisted wires from the composite was between 72.5 and 107 N, compared with 56.6 N for the braided wire. Furthermore, the average force required to deform the wire by 0.5 mm ranged from 0.62 to 1.84 N for twisted wires and 0.51 N for the braided wire [21].

Recent work by Seide et al. (2024) has provided some particularly interesting additional insights [23]. The authors demonstrated that when the wire–composite interface failed, even small horizontal forces were sufficient to cause the experimental teeth to rotate. This resulted in the wire strands tightening at the free end of the wire that was not covered by composite. Conversely, when the wire–composite interface remained intact, greater forces were needed to displace the teeth, resulting in the strands loosening at the free end of the uncovered wire [23].

The study by Labunet et al. (2022) confirmed the findings of Sifakakis et al. (2011) [18,22]. Using a stereoscopic microscope, the authors assessed the impact of physiological masticatory forces and bruxism on experimental splints. They found that the number of microcracks and cohesive detachments at the wire–composite interface was significantly higher in the presence of bruxism, regardless of the composite type used. However, flowable composites appeared to be particularly vulnerable [18,22].

Finally, it should be emphasized that masticatory and tongue forces can reach high levels in young and middle-aged adults. In a study by Kiliaridis et al. (1995), masticatory force at the incisors was found to be 113 N in men and 60 N in women [26]. Such forces are likely to induce tooth movement, particularly in the incisors. Similarly, Marín-Bernard et al. (2024) reported an average pressure of 49.6 kPa exerted by the anterior region of the tongue [27].

## 3. Proposed Etiopathogenic Model of WS in the Presence of a Multistrand Wire

In light of the results of the above-cited studies, as well as the clinical observations reported by several authors (Shaughnessy et al., 2016; Charavet et al., 2022, 2024; Abbas et al., 2024) and the international Severity-Based Classification of Wire Syndrome [4], it seems reasonable to consider WS to be a multifactorial phenomenon, the severity of which depends on the intensity and combination of the various factors involved in its initiation and progression [1,2,8,28]. In the context of a retainer using multistrand wires, several etiopathogenic hypotheses can be proposed.

### 3.1. Onset of WS

Wire syndrome may begin as soon as a point of failure appears in the fixed retainer [25]. This failure is thought to result primarily from the repeated mechanical stresses to which the retainer is subjected daily within the oral cavity.

The onset of the syndrome could be facilitated or even accelerated when the fixed retainer fabrication protocol is suboptimal, particularly in cases involving:

- Inaccurate adaptation of the wire to the tooth surfaces;
- Insufficient or uneven embedding of the wire in the composite;
- Inadequate length (too short or too long) of the free interdental segment;
- The composite posts having an unsuitable geometry.

Three scenarios can be envisaged in this context.

#### 3.1.1. First Scenario: Loss of Micromechanical Wire–Composite Interface

The composite posts and the free interdental segments of the wire in one or more teeth would be subjected to repeated stresses related to:

- Physiological dental movements;
- External stresses, such as nail-biting, teeth-grinding and oral hygiene practices;
- Internal stresses, such as occlusal trauma, chewing and swallowing.

These stresses generate compressive, tensile, shear and/or torsional forces. Due to their repetitive nature, these forces may lead to the progressive deformation of the free end of the wire. Initially, this deformation may be clinically imperceptible.

However, even minimal deformation of the free end of the wire could lead to microcracks forming within the composite posts. These microcracks would initially develop at the wire–composite interface, near the free end of the wire. Over time, these microcracks would gradually propagate along the wire–composite interface [6,19].

Ultimately, this process could result in the complete breakdown of the micromechanical interface between the wire and the composite without the composite detaching from the tooth surface or showing any visible fractures. Several in vitro studies confirm that this type of secondary alteration compromises the cohesion of the tooth–composite–wire assembly [29].

Consequently, the tooth could move around the axis of the wire released from the composite due to uncompensated horizontal muscular forces (lingual, mentalis and/or labial), which would cause an inclination in the direction of the dominant force. The tooth is no longer held in a stable position-it simply follows whichever muscular force is strongest.

**Figure 2:**
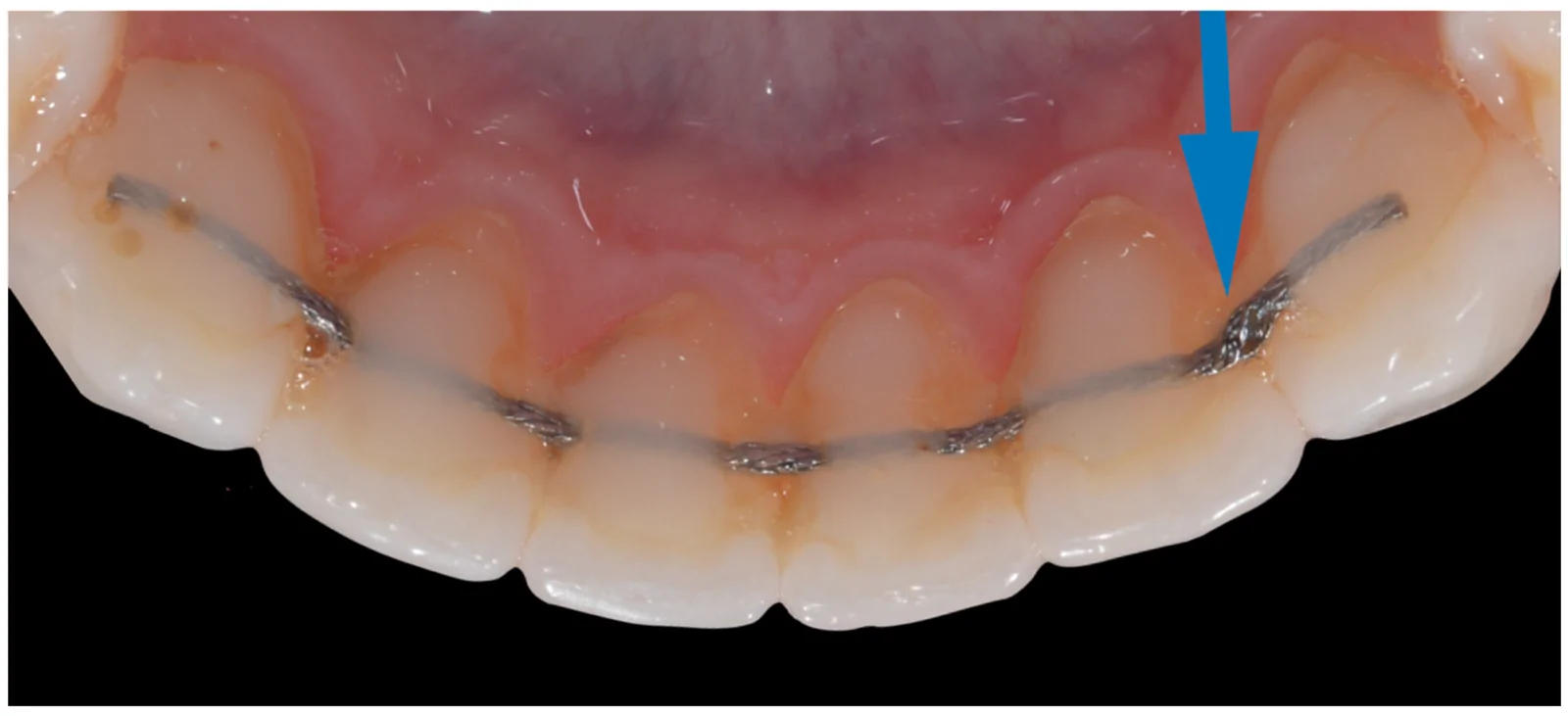
Interdental deformation of the wire between 33 and 32 (loosening). Photos courtesy of the authors: Prof. Sophie-Myriam Dridi and Prof. Carole Charavet.

**Figure 3:**
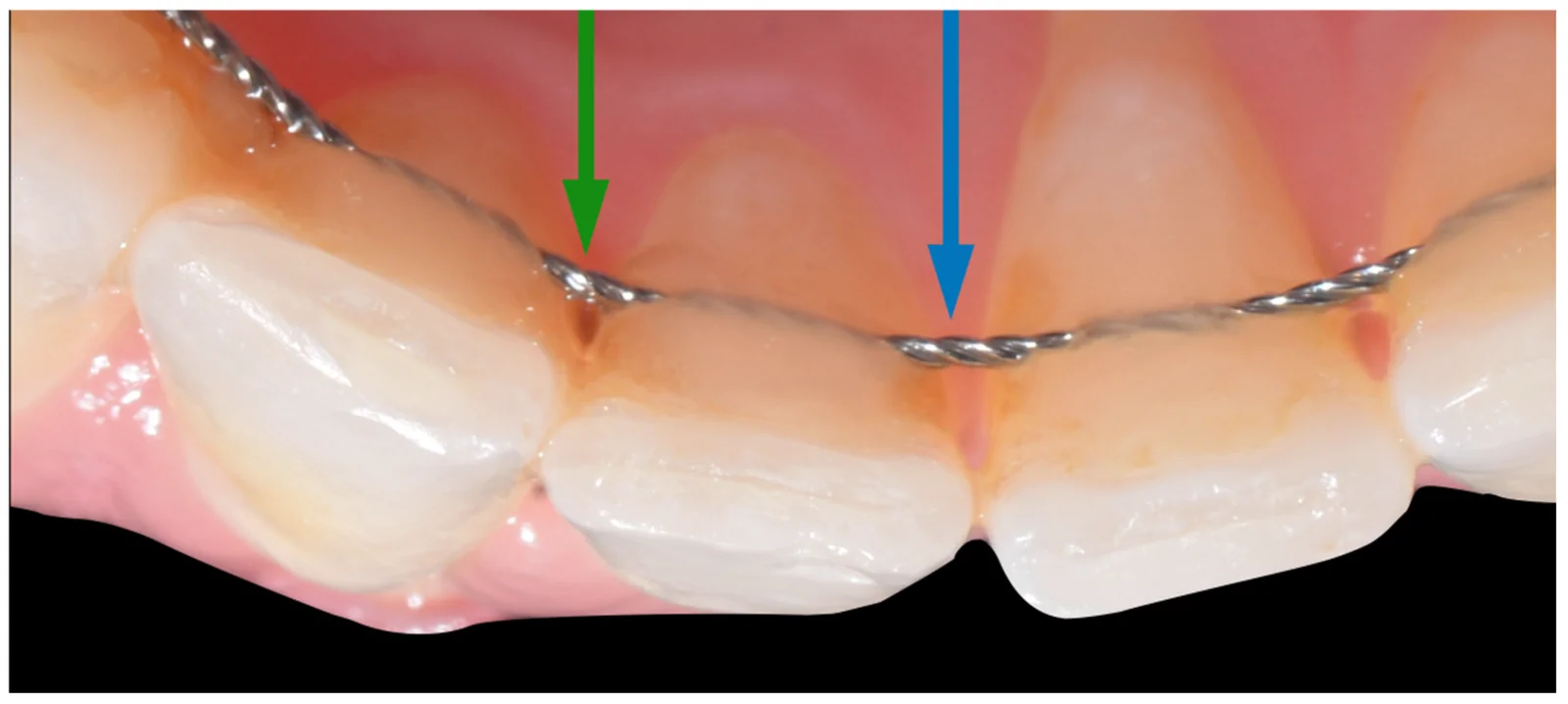
Interdental deformation of the wire between 42 and 41 (tightening) and between 41 and 31 (loosening). Photos courtesy of the authors: Prof. Sophie-Myriam Dridi and Prof. Carole Charavet.

**Figure 4:**
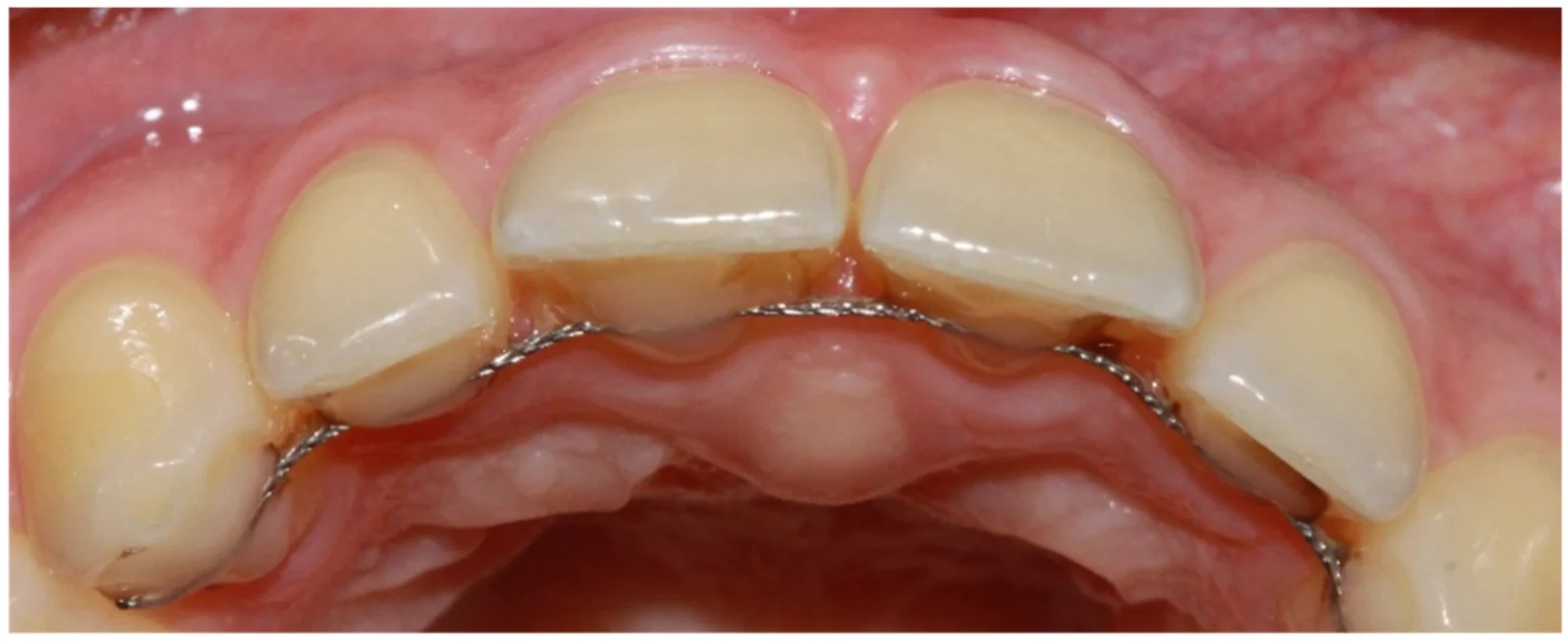
Loosening of the strands between the central incisors and mesial tightening of the canines. Photos courtesy of the authors: Prof. Sophie-Myriam Dridi and Prof. Carole Charavet.

For the intermediate teeth in the fixed retainer that are constrained mesially and distally, the tooth–composite post assembly could pivot on the buccal side or the opposite side around the wire, with variable resistance depending on the residual helical impression left by the wire within the composite on its inner wall. Where this resistance persists, the wire would undergo additional reciprocal stress, promoting increased deformation of the interdental segments. This deformation would gradually alter the wire’s mechanical properties, leading to secondary activation of the fixed retainer comparable to an orthodontist-shaped orthodontic wire (Figure 2, Figure 3 and Figure 4).

**Figure 5:**
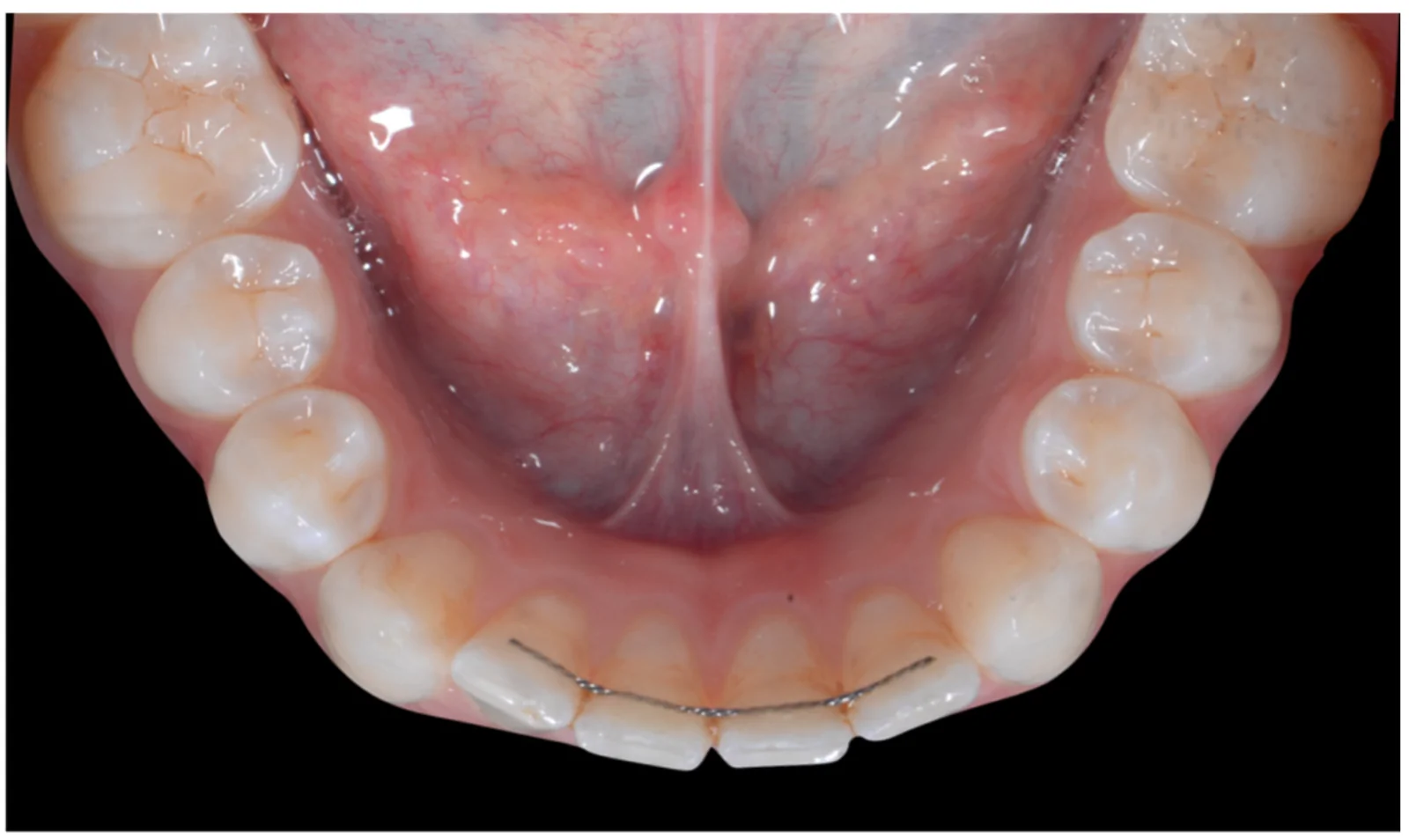
Retention cut distally at 42 and 32, disto-lingual rotation of 42. Photos courtesy of the authors: Prof. Sophie-Myriam Dridi and Prof. Carole Charavet.

In the case of terminal teeth in retention that are not distally confined, the tooth may be more prone to rotation and tooth inclination (Figure 5) [30]. This type of movement could be explained by the wire slipping within the composite, particularly if its ends are not folded back and/or the composite coating is compromised (e.g., thinned by abrasion or cracked/fractured).

#### 3.1.2. Second Scenario: Maintenance of Micromechanical Wire–Composite Adhesion

**Figure 6:**
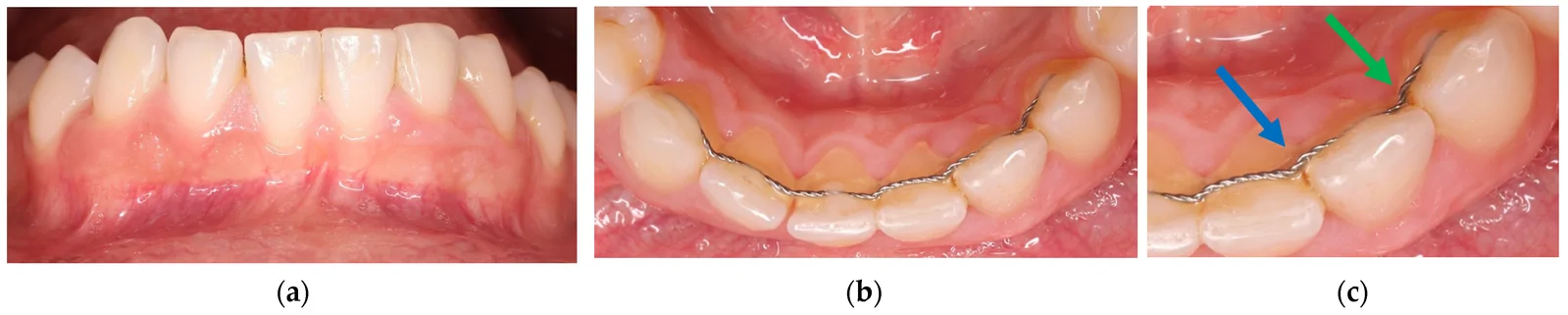
Wire syndrome in the incisors, which exhibit differential torques, (**a**) with the onset of a diastema. (**b**) Loosening of the strands between the incisors and tightening between 32 and 33. (**c**) Patient presenting with lingual dysfunction (low tongue resting mainly on 31, 41 and 42). Photos courtesy of the authors: Prof. Sophie-Myriam Dridi and Prof. Carole Charavet.

Despite the daily stresses exerted on the fixed retainer, the wire–composite interface would remain intact at the retained teeth. However, when subjected to a powerful muscular force, the teeth would move while still being held by the retention wire (Figure 6).

This would generate high stresses at the free ends of the wire, causing deformation in response to tooth movement [23]. The type of deformation observed-tightening or loosening of the strands-depends on:

- The type of wire used;
- The extent of tooth movement;
- As well as the forces exerted on the wire.

As in the first scenario, this deformation would gradually alter the wire’s mechanical properties and lead to the retainer becoming activated again.

Significant wire deformations are particularly evident in cases of lingual thrust, where the strands untwist or tighten.

#### 3.1.3. Third Scenario: Mixed Mechanism

All of the above phenomena could occur simultaneously within the same fixed retainer. Therefore, cohesive failure of the wire–composite interface could be observed in some areas, while adhesion would remain intact in others.

This mixed mechanism could help to explain the wide variability in the clinical manifestations of wire syndrome.

### 3.2. Progression of WS

The progression of WS is generally gradual and insidious, extending over several months or even years [1].

In vitro tensile and flexural tests support this hypothesis by demonstrating that, up to a certain threshold, most multistrand metal wires exhibit high tensile strength while retaining significant deformability [24]. In clinical practice, patients usually seek consultation when the wire has broken or the composite has come loose. This may explain why the initial stages of WS often go unnoticed by both patients and practitioners.

Over time, the cumulative effects of external and/or internal stresses exerted on the retainer could progressively worsen the clinical presentation and its tissue consequences.

Rupture of the wire–composite interface, with or without persistence of the helical impression within the composite, could spread to other teeth not initially affected by WS.

**Figure 7:**
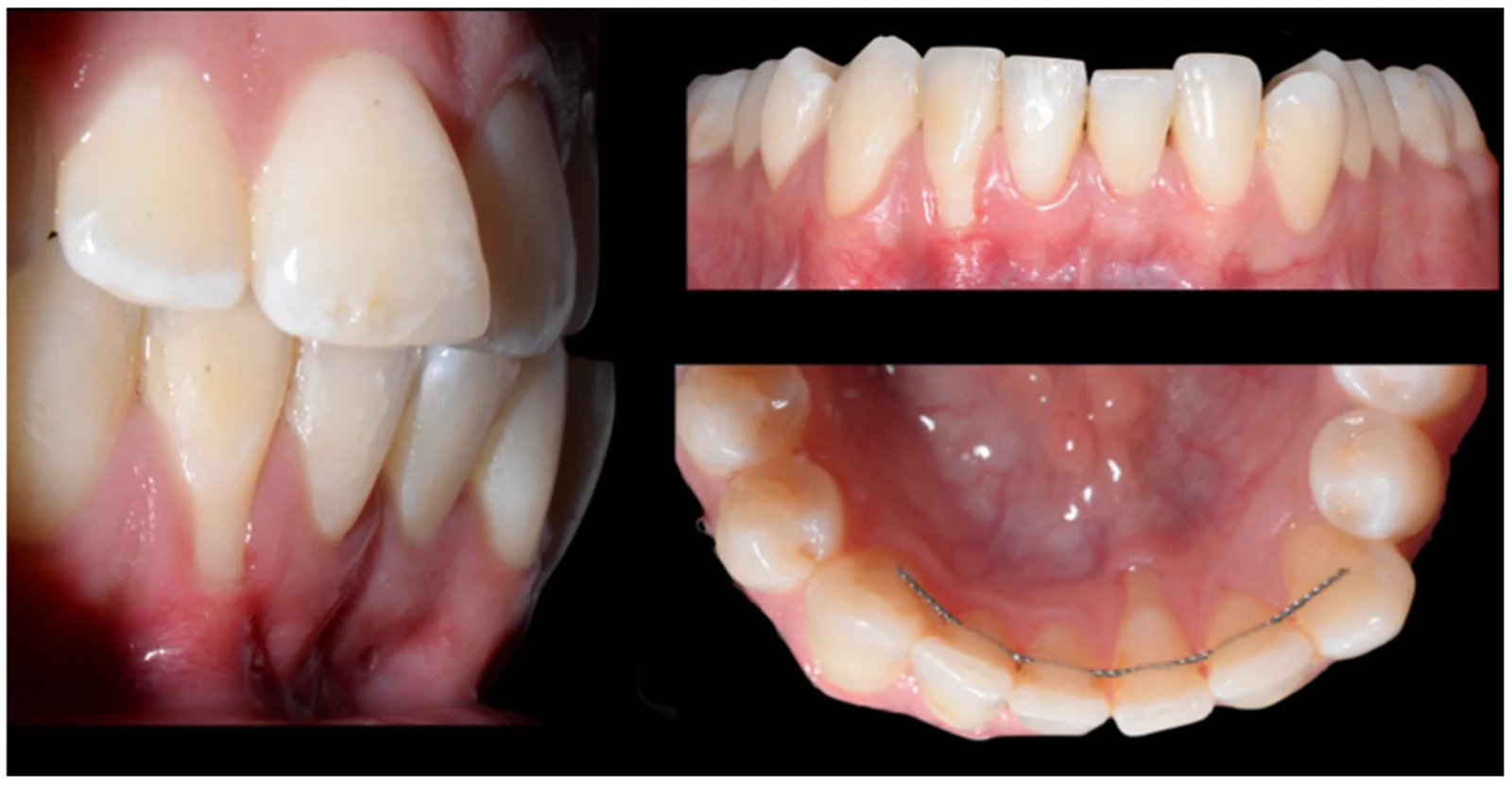
Fixed retainer in place for several years. Presence of an incisor ‘X-effect’ associated with a canine ‘twist-effect’. Photos courtesy of the authors: Prof. Sophie-Myriam Dridi and Prof. Carole Charavet.

**Figure 8:**
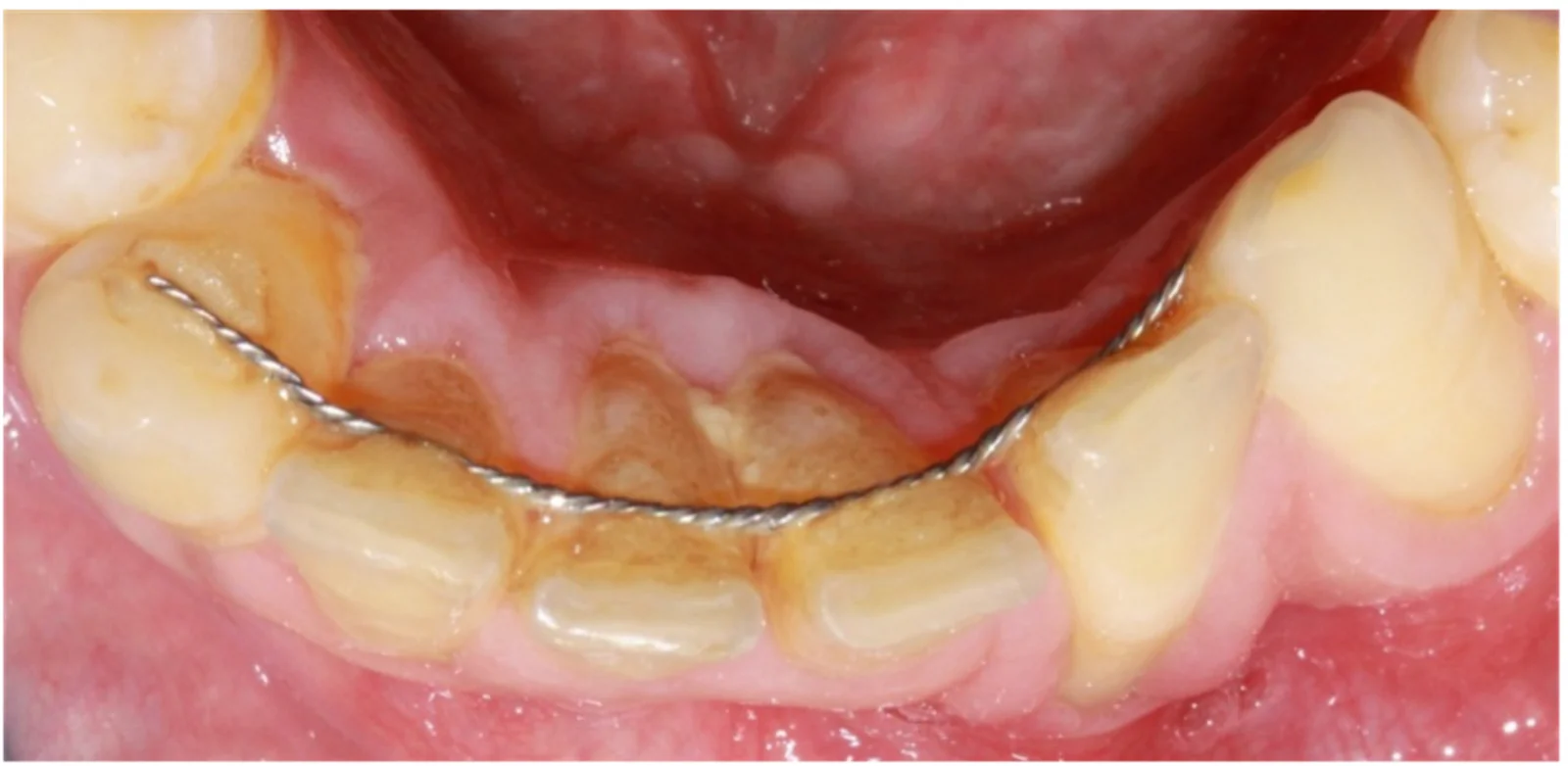
Long-standing fixed retainer associated with an incisor ‘X-effect’ and a canine ‘twist-effect’. Photos courtesy of the authors: Prof. Sophie-Myriam Dridi and Prof. Carole Charavet.

**Figure 9:**
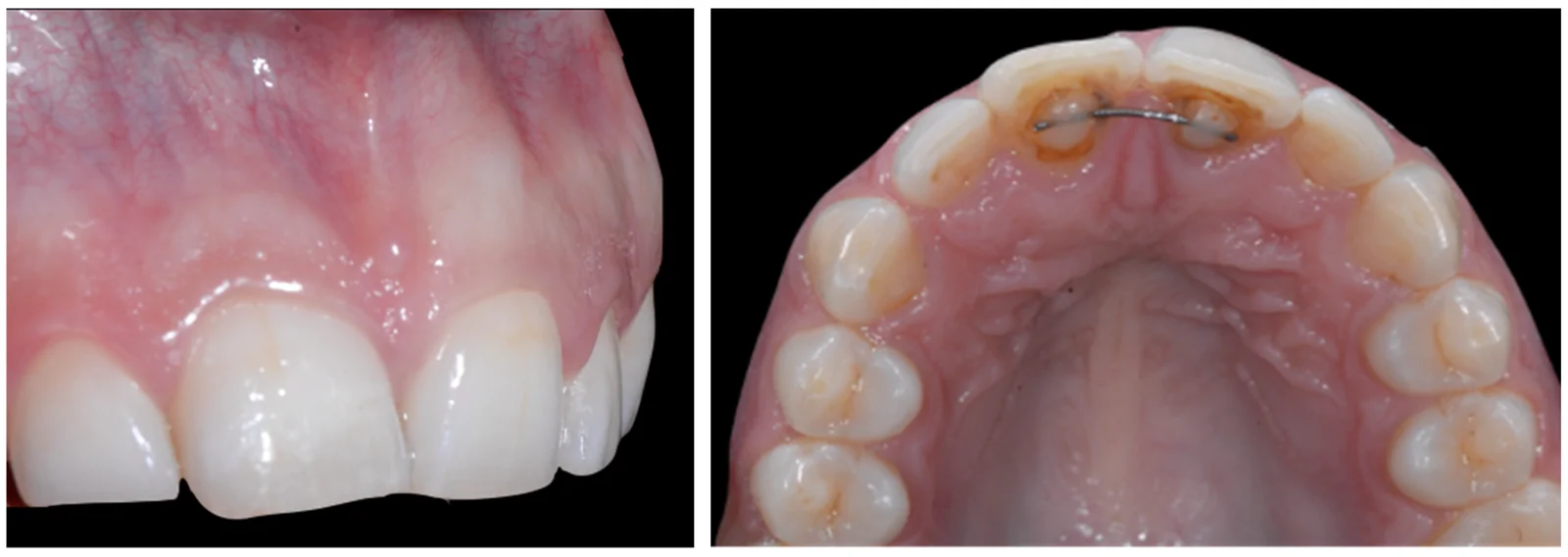
Incisal X-effect [3]. Photos courtesy of the authors: Prof. Sophie-Myriam Dridi and Prof. Carole Charavet.

These teeth would then tilt in a direction dependent on the nature of the forces involved, as well as the type of wire used (Figure 7, Figure 8 and Figure 9). Initially, this process may correspond to an attempt at mechanical rebalancing of the forces applied to the teeth. Clinically, this development would lead, in particular, to:

- The ‘X-effect’ observed in the incisors;
- The ‘twist-effect’ observed in the canines.

**Figure 10:**
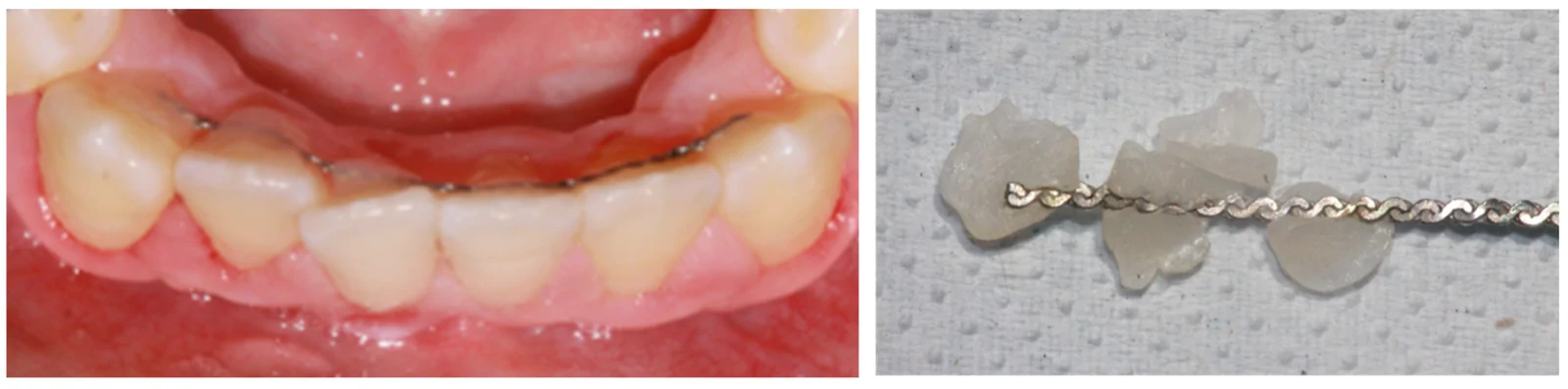
Fixed retainer in place for several months. Detachment of the composite at tooth 41, which, according to the patient, appeared two days prior to the consultation. The lingual displacement of tooth 42, which is still maintained, is associated with insufficient coating of the wire and probable incipient metal corrosion in a context of gingival inflammation. Photos courtesy of the authors: Prof. Sophie-Myriam Dridi and Prof. Carole Charavet.

**Figure 11:**
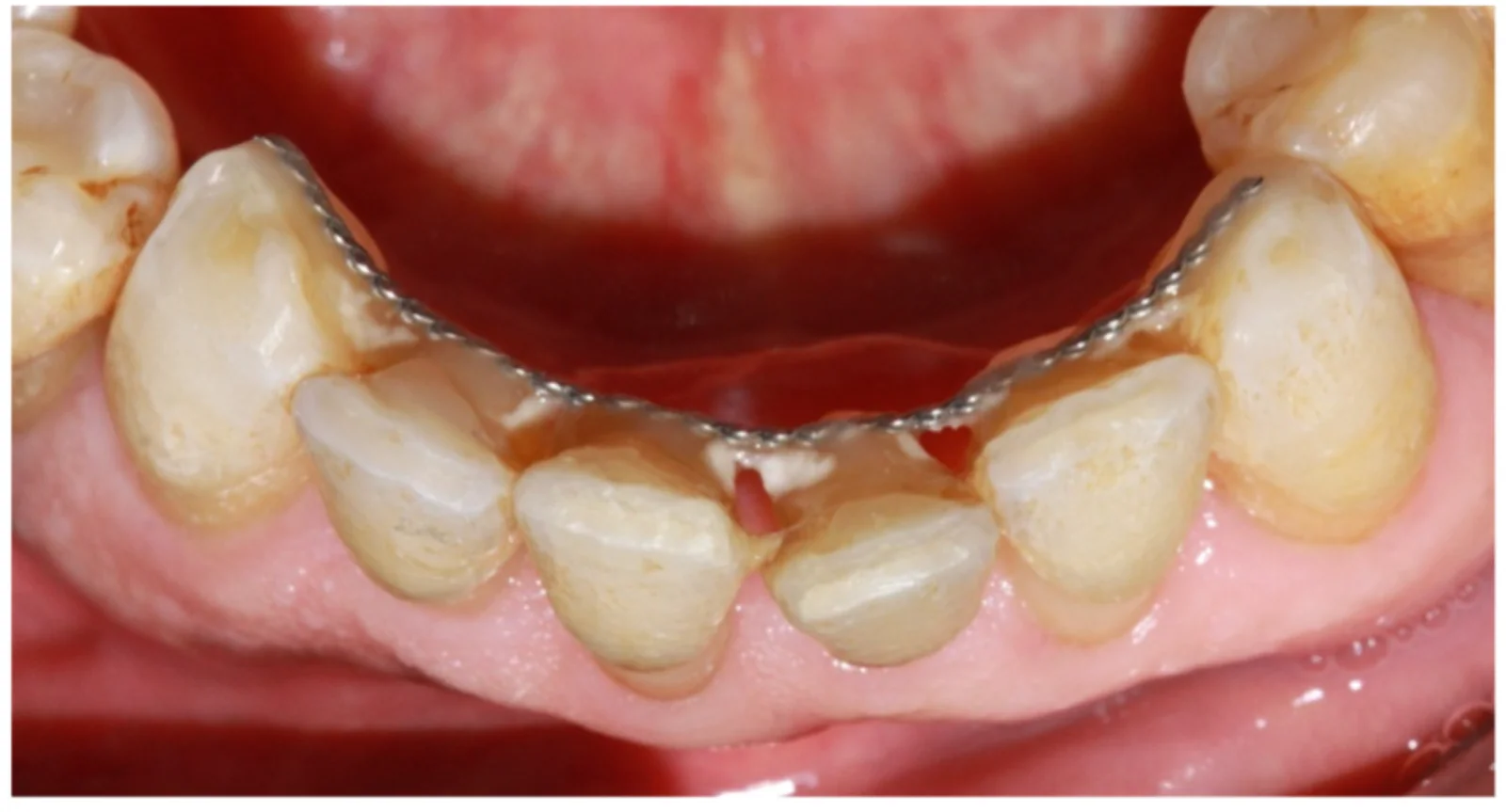
Old fixed retainer with composite fracture at tooth 42. The wire appears poorly fitted to the lingual surfaces and poorly embedded in the composite. Presence of tartar on the retainer wire. Photos courtesy of the authors: Prof. Sophie-Myriam Dridi and Prof. Carole Charavet.

**Figure 12:**
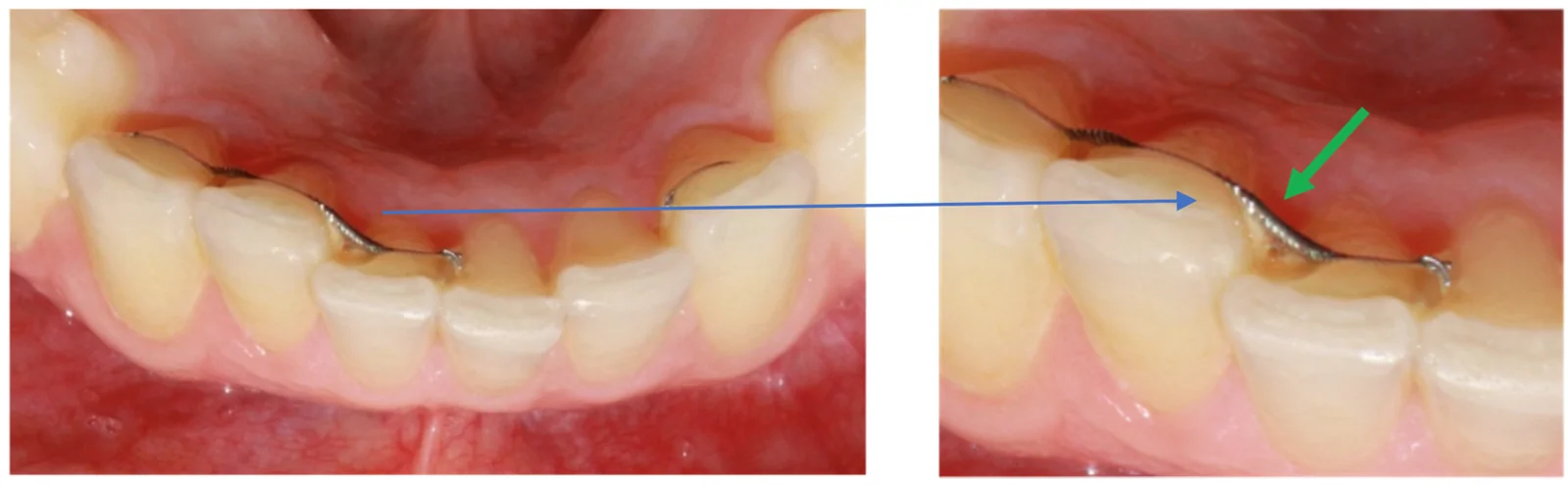
Fixed retainer in place for years. Fracture of the fixed retainer wire between 31–41 and 32–33, associated with detachment of the composite posts. Tightening of the strands observed at the free segment between 42 and 41. Photos courtesy of the authors: Prof. Sophie-Myriam Dridi and Prof. Carole Charavet.

Progressive worsening of WS could exacerbate deformation of the free wire segments to a critical point, marked by either wire fracture or detachment of the composite from tooth surfaces (Figure 10, Figure 11 and Figure 12). These are two complications that are frequently reported in clinical practice [15].

The severity of this phenomenon may be exacerbated by:

- Inadequate design of the fixed retainer;
- Abrasion of the composite posts [16];
- Electrochemical corrosion of the wires, which can affect their mechanical properties [31];
- The accumulation of dental plaque or the presence of gingival inflammation (Figure 10).

**Figure 13:**
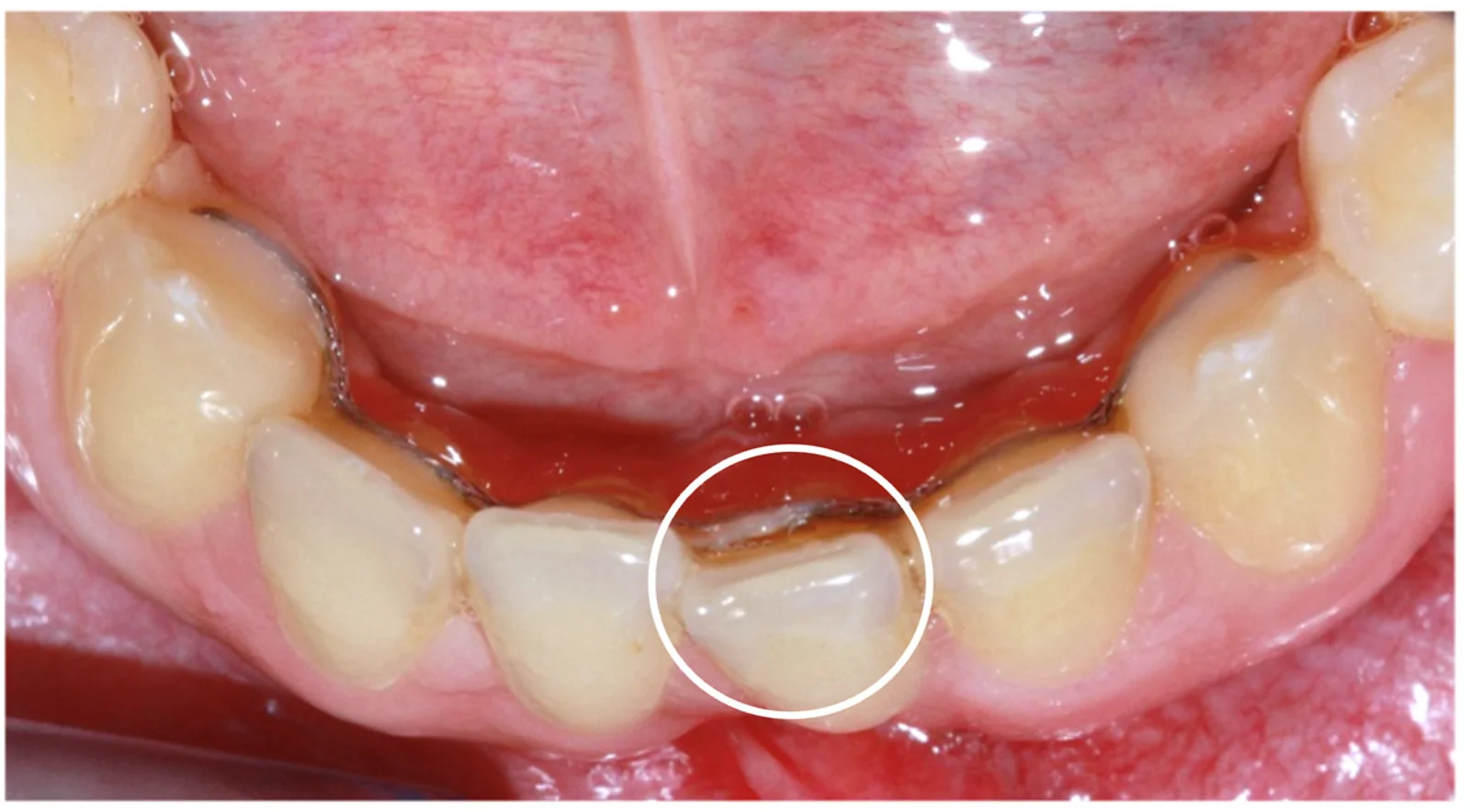
Detachment of the wire from the composite on tooth 31. According to the patient, this failure occurred within a month of the retainer being fitted. Photos courtesy of the authors: Prof. Sophie-Myriam Dridi and Prof. Carole Charavet.

In situations where the wire–composite bond remains intact on teeth that were not initially affected by WS, mechanical stresses could lead to interdental wire fracture and/or composite detachment from the tooth surface more prematurely (Figure 13).

## 4. Clinical Implications and Prevention

This section was written based on the authors’ clinical expertise, their review of the published literature in this field, and their own clinical and fundamental research. A systematic review and meta-analysis should be conducted to confirm and further strengthen these findings.

Wire syndrome is likely to be underdiagnosed in everyday practice. Its slow progression and asymptomatic nature in the early stages mean that the initial clinical signs are often overlooked by both patients and practitioners. However, the severe dental, periodontal and esthetic consequences of WS raise a potential medico-legal issue, as it may be perceived as a complication of orthodontic treatment.

Against the backdrop of a steady increase in the number of adult patients undergoing orthodontic treatment-and consequently, the use of long-term fixed retainers-the incidence of WS could rise in the coming years. This trend highlights the critical importance of post-orthodontic follow-up, which is still too often neglected in clinical practice.

In light of the currently available data, clinical recommendations should probably not focus solely on the choice of retainer wire, as no scientific evidence has yet demonstrated the superiority of any specific fixed retainer in preventing WS. Therefore, the clinician’s attention should be directed towards the rigor of the clinical protocol for fitting the retainer.

Therefore, the placement of a fixed retainer cannot be considered a quick or secondary procedure. It requires a precise and reproducible protocol, the quality of which largely depends on the practitioner’s experience [32]. Rigorous isolation of the surgical field to prevent saliva contamination is essential for optimal adhesion between enamel, composite, and metal wire.

To achieve positioning of the wire that is as precise, passive and reproducible as possible, the placement of the retention wire via indirect bonding using a transfer tray may also be recommended.

The retention wire must fit the tooth surfaces perfectly and be correctly embedded within the composite posts. However, the posts should not cover the lingual or palatal surfaces of the teeth excessively to preserve their physiological micro-movements. The geometry, thickness and position of the composite posts, as well as the distance between the interdental posts, are important parameters to consider.

Bonding the posts as far as possible from the gingival margin could also facilitate oral hygiene and limit associated periodontal complications [33].

Furthermore, the data analyzed in this review suggest that WS should be viewed as a dynamic and evolving phenomenon. Depending on the clinical conditions, such as the type of retainer used, dental morphology, the periodontal context, the presence of plaque or tartar, gingival inflammation, composite abrasion or the extent of functional stresses, the process may gradually progress from the initial formation of WS to wire fracture or composite detachment.

Two clinical scenarios therefore appear possible:

1. Either the initial retention protocol was inadequate, with the wire insufficiently or incorrectly embedded in the composite;
2. Or the fixed retainer was initially correctly fabricated but subsequently subjected to excessive mechanical stress.

In this context, regular and prolonged monitoring is essential to detect any wire deformation, composite deterioration or abnormal tooth movement early on. Patient information and education also play a central role, particularly with regard to oral hygiene, parafunctional habits, and the necessity of long-term follow-up.

Finally, given the potential mechanisms involved in wire syndrome, repairing or re-bonding an already compromised retention wire should probably be avoided, as these procedures may alter the fixed retainer’s initial mechanical properties and trigger WS. Based on our expertise, removal appears to be the appropriate course of action in cases of progressive tooth movement and/or periodontal lesions, wire deformation, composite deterioration, or a second instance of wire detachment.

These clinical requirements necessitate a re-evaluation of the time spent on retainers, as well as the associated costs of fabrication and maintenance.

## 5. Future Perspectives

To date, no comparative study has demonstrated the superiority of any specific fixed retainer in preventing WS. Consequently, there is currently no consensus on the most suitable type of fixed retainer or the most effective clinical protocols for minimizing the risk of this syndrome [34].

In clinical practice, the choice of retention system is primarily based on the practitioner’s personal experience and professional habits, as well as the characteristics of the initial malocclusion [35,36].

In this context, further studies providing a high level of evidence in both in vitro and in vivo settings appear essential to improving clinical protocols and optimizing the prevention of WS, even if their implementation remains methodologically complex and costly [34].

Artificial intelligence (AI) could be a promising approach to studying and preventing WS. In particular, the automated analysis of clinical images, digital models or biomechanical data could enable the following: early detection of retainer wire deformations. For example, AI could be used to automatically compare successive optical impressions taken during post-orthodontic follow-up. This type of analysis could detect minimal tooth movements consistent with the onset of WS early on, sometimes even before visible clinical signs or symptoms are reported by the patient.

This would improve:

- The early diagnosis and longitudinal monitoring of retention appliances;
- The identification of individual risk factors;
- The prediction of situations with a high risk of retainer failure.

Furthermore, machine learning-based approaches could contribute to the development of personalized retention protocols tailored to each patient’s specific biomechanical and functional characteristics.

## 6. Limitations

The limitations of this narrative review are inherent in its design, as it is not based on a systematic methodology. This synthesis is based on a qualitative analysis of a limited number of mainly in vitro studies characterized by heterogeneous protocols, rather than prospective studies, which currently do not exist but should be developed. Nevertheless, this review provides an exploratory synthesis of WS, a condition about which little is currently known, with the aim of identifying avenues of research that could shed light on the mechanisms underlying its onset.

## 7. Conclusions

These findings confirm that wire syndrome should be regarded as a complex, multifactorial complication of bonded fixed orthodontic retainers, requiring a structured diagnostic and preventive approach in clinical practice.

The currently available clinical and experimental data suggest that it develops as a result of the dynamic interactions between the retainer’s biomechanical properties, the quality of the clinical bonding protocol and the functional stresses exerted within the oral environment.

In the case of multistrand wires, it appears that the wire–composite interface plays a central role in initiating the pathological process.

**Figure 14:**
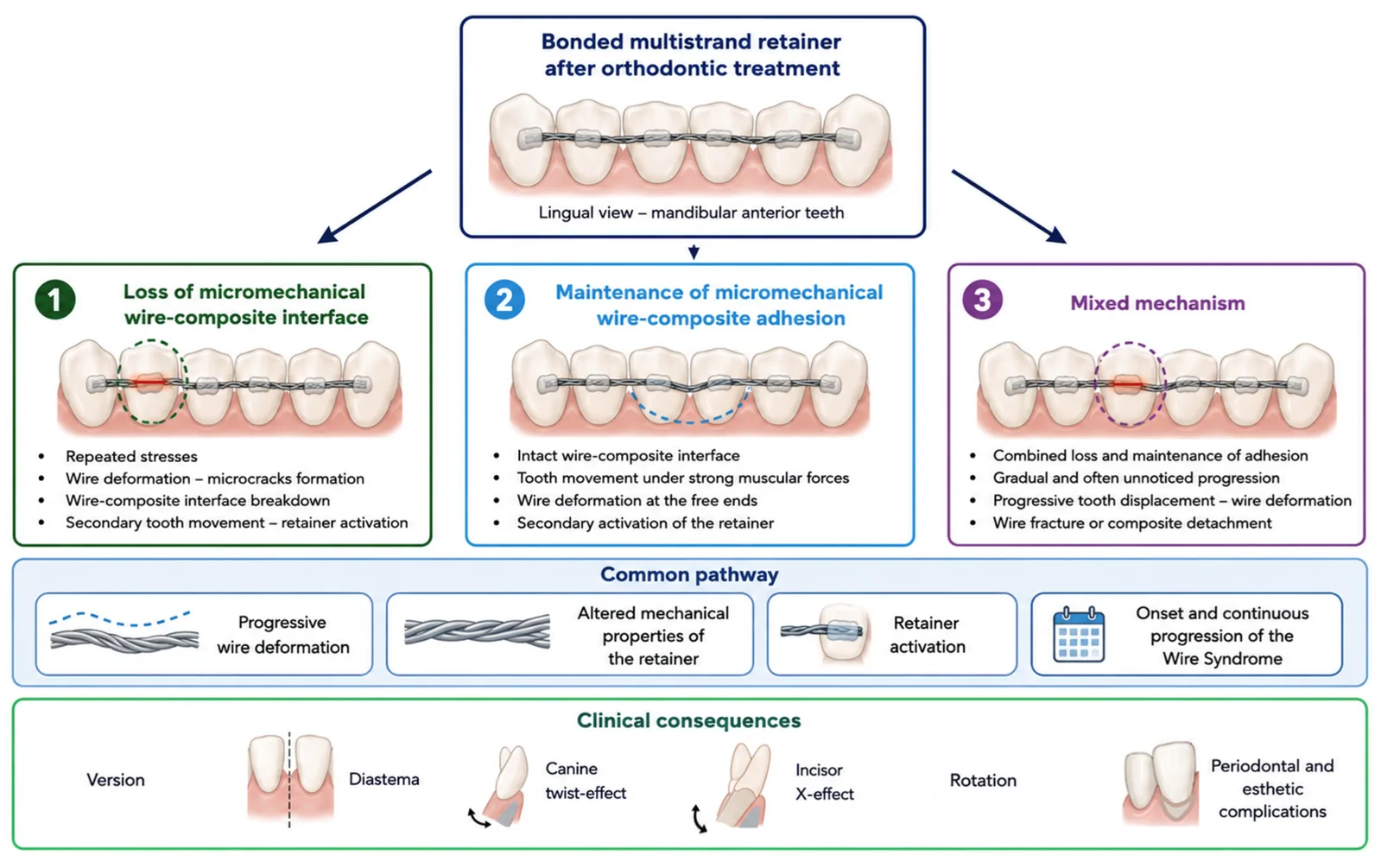
Wire syndrome associated with multistrand orthodontic retainers: a proposed etiopathogenic concept. Diagram by the authors: Prof. Sophie-Myriam Dridi and Prof. Carole Charavet.

This narrative review therefore proposes several etiopathogenic hypotheses that could explain the wide clinical variability and often slow, insidious progression of WS (Figure 14). It also emphasizes the importance of prevention. Further high-quality studies are needed to improve our understanding of the mechanisms of wire syndrome, identify patients at risk and optimize clinical retention protocols.

## Figures and Tables

**Table 1:** In vitro tests.

Authors	Materials Tested	Methodology	Main Results	Implications for the Fiber/Composite Interface
**Bearn et al. (1997)** [16]	Two three-stranded (0.0175 inch) and five six-stranded coaxial wires (0.0175, 0.018 or 0.0215 inch). Six types of composites.	1. **Tensile test** - 5 acrylic cylinders for each type of wire, each with a comparable hole (3 mm × 2 mm) drilled into its surface, into which the end of a composite-coated wire is inserted - Tensile test along the longitudinal axis of the wire until it breaks 2. **Scanning electron microscopy analysis** - Visual analysis of composite residues on the surface of the torn fibers 3. **Tensile test according to composite thickness and abrasion test** - 5 acrylic cylinders per composite type - Insertion of a filament embedded in composite into grooves of varying depths machined into the surface of the cylinders, followed by welding of both ends of the filament so that it can be attached to a hook fixed to the tensile load cell of the testing machine, then tensile testing - Abrasion testing of the various composites	1. No significant difference was detected between different multistrand wire types and diameters regarding retention in composite. The pull-out force varied on average from 58.7 to 85.6 N. 2. After pull-out, the three-strand wires exhibited the largest composite debris on their surface, which was distributed at regular intervals. The six-strand wires exhibited a significant amount of small composite fragments (better retention). 3. The deeper the filaments were embedded in the composites, the greater the pull-out force. 4. Abrasion resistance varied depending on the composite.	The surface characteristics of a retention wire influence its adhesion to the composite. Composite wear can lead to failure of the retainer.
**Cooke and Sherriff (2010)** [17]	Two three-stranded wires (0.016 × 0.022, and 0.0175 inches). Standard composite.	1. **Flexural test** - 26 pairs of human incisors inserted into specific acrylic molds and connected with a wire encased in two composite blocks (4 mm × 1.5 mm), 2 groups per wire: 1st test then 2nd test after retrieval of the detached wires - Vertical force applied to the mid-interdental portion of the wire until the wire detaches or breaks 2. **Analysis of composite residues using a stereomicroscope** - Assessment of the ARI (adhesive remnant index) to determine the type of failure (adhesive, cohesive, or both) - Visual analysis of wire deformation	1. Regarding the force required to break the wire, there was no significant difference depending on the type of wire in either test. This force was lower in the 2nd test (averaging between 37 and 41 N in the 1st test versus 14 N in the 2nd test). 2. The type of fracture was comparable for both types of wire. In the 1st test, there were only cohesive fractures for both types of wire, with comparable rates. In the 2nd test, there were adhesive fractures, but in reduced numbers for both types of wire. The deflection of the wires was comparable and of the same magnitude during both bonding sessions. No fractures were observed for either type of wire.	When the retainer is subjected to a vertical force, the free end of the wire undergoes stresses that cause its deformation and ultimately a cohesive fracture. Reusing a detached wire reduces its mechanical properties.
**Sifakakis et al. (2011)** [18]	A 3-strand twisted wire (0.0195 inch) and two 6-strand twisted wires (0.0215 inch). Standard fluid composite	1. **Deformation test** - 10 specimens per wire type - Each specimen consisted of 6 acrylic teeth (canine to canine) connected by a wire embedded in composite within a lingual groove (2 mm × 2 mm); the distance of the unembedded wire between the teeth was 4 mm - The specimens were designed so that only a lateral incisor could move under the effect of a controlled force - Application of a vertical force and a horizontal force to this incisor until a displacement of the free wire of 0.2 mm was achieved in increments of 0.02 mm	1. For a comparable degree of tooth movement, the horizontal force was always greater than the vertical force. 2. Simulated intrusion-extrusion and buccolingual movements of 0.2 mm generated forces on the teeth of approximately 1 N in the vertical direction and 1.5 N in the horizontal direction (no significant difference for the three wires).	The determining factor to be considered is the extent of the wire movement rather than its intrinsic properties. Forces ≥ 1 N can cause tooth movement.
**Baysal et al. (2012)** [19]	A five-strand twisted cable (0.0215 inch), a flexible, eight-strand rectangular braided (0.016 × 0.022 inch) and a soft coaxial wire (0.0195 inch). Standard composite.	1. **Bending test** - 47 pairs of human incisors set in acrylic molds and connected with a wire encased in two composite posts (4 mm × 1.5 mm), with one group per type of wire - Vertical force applied to the mid-interdental portion of the wire 2. **Analysis of composite residues using a stereomicroscope** - Assessment of the ARI (adhesive remnant index) to determine the type of fracture (adhesive, cohesive, or both) - Visual analysis of wire deformation 3. **Tensile test** - 3 groups of 20 acrylic cylinders, each with a comparable hole (3 × 2 mm) drilled into their surface, into which the end of a composite-coated wire is inserted - Tensile test along the longitudinal axis of the wire until it breaks	1. There was no statistically significant difference in the pull-out force between the wires tested. 2. All three types of failure were observed for all three types of wire. The flexible wires deformed significantly more than the 5-strand twisted wire (the extent of deformation was comparable between the flexible wires). 3. The pull-off force was significantly lower for the flexible wires (35 and 37.9 N) compared to the 5-strand twisted wire (74.6 N).	The deformation of the wire sections not covered by composite depends on the nature of the wire and the magnitude of the forces exerted on the wire.
**Paolone et al. (2015)** [20]	A straight wire (0.016 × 0.022 inch), two gold round twisted wires (0.0215 inch), a rectangular braided wire (0.016 × 0.022 inch). Two micro-hybrid composites, one micro-nano-filled composite.	**Tensile test** - 10 samples per wire/composite combination - Each sample consisted of a composite tube 3.5 mm in diameter, into the center of which a retention wire was inserted during bonding - Tensile test along the longitudinal axis of the wire until it broke	The best retention was achieved with gold-plated twisted wires or rectangular braided wire and the micro-nano-filled composite.	The adhesion of the wire to the composite depends on the combined mechanical properties of the two wire–composite components.
**Milheiro et al. (2015)** [6]	Two flexible twisted strands (3 and 6 strands), two rigid twisted strands (6 strands) + two types of glass fiber. Standard composite.	1. **Three-point bending test:** - Assessment of the type of fracture under bending: adhesive (enamel/composite), cohesive (wire/composite), or a combination of both - 10 samples per clamping retainer - Each sample consisted of 3 bovine enamel discs, spaced 6.5 mm apart, connected by a wire embedded in approximately 1 mm of composite bonded to the surface of the discs. The size of the composite blocks varied between 6, 4 or 2 mm. The discs were mounted on specialized fixtures to allow for a 3-point bending test 2. **Finite element analysis models** - Analysis of the dynamic behavior of retainer composed of twisted wire	1. The best resistance to deformation and fracture was obtained with flexible wires. Glass fibers performed the worst. 2. The failure mode was generally cohesive. The bond strength was better when the composite pads were 4 and 2 mm thick.	The wire/composite interface plays a major role in the strength of the retainers. The most critical area of these retainers is the wire–composite interface near the free end of the wire, rather than the enamel–composite interface. The length of the free wire between the composite posts influences the distribution of stresses at the wire–composite interface.
**Samson et al. (2018)** [21]	Two 3-strand twisted wires (0.030 and 0.036 inch) and one 3-strand braided wire (0.016 × 0.022 inch). Standard composite.	**Tensile test and 3-point bending test** - 3 groups of 20 specimens per wire type - Each sample consisted of a pair of human incisors inserted into specific acrylic molds and connected with a wire embedded in composite blocks (4 mm × 1.5 mm) - A vertical force was applied to the mid-interdental portion of the wire until a deflection of 0.5 mm was achieved and the wire broke.	1. The average adhesive force required to cause the wire to peel off ranged from 107 to 72.5 N for twisted wires and was 56.6 N for the braided wire. 2. The average force required to cause a 0.5 mm deformation of the wire ranged from 1.84 to 0.62 N for twisted wires and was 0.51 N for the braided wire.	The deformation of the wire sections not covered by composite depends on the nature of the wire and the magnitude of the vertical force exerted on the wire.
**Labunet et al. (2022)** [22]	A 6-strand twisted round wire. Standard fluid or nano-hybrid composite	**Analysis of composite residues using a stereomicroscope** - 40 samples consisting of 2 human teeth, embedded in a retainer simulating periodontal tissue and connected by a wire encased in a composite post - The samples were subjected to physiological masticatory cycles or cycles simulating bruxism using a specific testing machine - 4 groups of 10 samples according to composite type and cycle type - Assessment of the ARI (Adhesive Remnant Index) to determine the type of fracture (adhesive, cohesive, or both)	1. The number of microcracks within the composite was significantly higher in cases of bruxism. Cohesive fractures were observed at the wire–composite interface in cases of bruxism. 2. The flowable composite proved to be less resistant and more vulnerable than the hybrid composite, but only in cases of bruxism (significantly higher number of microcracks).	Excessive masticatory forces generate numerous microcracks within the composite and weaken the wire–composite interface.
**Seide et al. (2024)** [23]	A 3-strand round twisted wire with different pre-treatments prior to placement. Standard flowable composite.	**Tensile test:** - 4 groups of 20 samples - Each sample consists of two composite discs, mounted on a specific support, connected by a retaining wire with a 3 mm interdental section not covered by composite - Modification of the wire–composite interface on one of the discs: group 1 = wire coated with Vaseline (no wire–composite adhesion), group 2 = no pre-treatment, group 3 = degreasing with ethanol (strong wire–composite adhesion), group 4 = degreasing with ethanol + wire ends bent - Artificial aging for half of the retainers (simulation of a two-year retention period in an oral environment) - Horizontal force applied to increase the inter-disc distance in 0.01 mm increments and induce stresses in the wire	1. In Group 1, with an isolated composite–wire interface, the application of low horizontal tensile forces caused the test disc to rotate in the direction of the coils, with the strands tightening at the interdental portion of the wire (torque of 1.7 N mm for a force of 10 N). For the other groups, with an adherent wire–composite interface, forces almost twice as high were required to cause a rotation of the test disc in the opposite direction to the coils, with loosening of the strands at the interdental portion of the wire (torque of −0.5 N mm for a force of 18 N; significantly lower rotational moments for these groups). 2. Artificial aging of the retainers reduced the wires’ resistance to deformation. 3. Retainers with folded wire ends proved to be more resistant even when subjected to artificial aging.	Wire preparation directly influences the quality of the wire–composite bond and, ultimately, its resistance to deformation and the type of deformation when the wire is subjected to stress. The deformation of the wire sections not covered by composite depends on the wire preparation and the magnitude of the horizontal force exerted on the wire.
**Kurnaz et al.** **(2025)** [24]	Seven solid CAD/CAM round titanium-nickel wires (0.018 × 0.018 inch), with different surface treatments: no treatment, electropolishing, laser texturing, and plasma treatment. A 3-strand twisted stainless steel wire (0.018 inch) with no surface treatment. Standard composite.	**Bending test** - 7 groups of 8 samples per wire type - Each sample consists of a pair of human incisors inserted into specific acrylic molds and connected with a wire encased in composite - Applying a vertical force to the mid-interdental section of the wire until it bent and detached	1. The average detachment force varied between groups from 77.92 N to 132.99 N. Titanium-based wires proved to be more resistant. Surface treatment involving an electropolishing process first, followed by laser texturing and atmospheric plasma treatment, yielded the best results. However, there was no statistical difference between the groups. 2. The average elongation values ranged from 0.7 mm to 2.01 mm. The stainless-steel wire exhibited higher elongation values than the other wires, with a significant result.	Stainless steel wire is more flexible than titanium-based wires, which is an advantage in the presence of high occlusal forces and periodontal problems. Titanium-based wires with a surface treatment are less flexible but more resistant to detachment.

## Data Availability

No new data were created or analyzed in this study. Data sharing is not applicable to this article.

## Abbreviations

The following abbreviations are used in this manuscript:

WS	Wire Syndrome
AI	Artificial intelligence
ARI	Adhesive Remnant Index
